# Correction: Reanalysis of the 2000 Rift Valley fever outbreak in Southwestern Arabia

**DOI:** 10.1371/journal.pone.0248462

**Published:** 2021-03-08

**Authors:** Compton J. Tucker, Katherine A. Melocik, Assaf Anyamba, Kenneth J. Linthicum, Shamsudeen F. Fagbo, Jennifer L. Small

The Funding statement is incorrect. The correct Funding statement is as follows: A.A: This project was funded by the Armed Forces Health Surveillance Division, Global Emerging Infections Surveillance (GEIS) Branch, ProMIS ID P0072_19_NS.

## References

[1] Tucker CJ, Melocik KA, Anyamba A, Linthicum KJ, Fagbo SF, Small JL (2020) Reanalysis of the 2000 Rift Valley fever outbreak in Southwestern Arabia. PLoS ONE 15(12): e0233279. 10.1371/journal.pone.0233279 33315866 PMC7735616

