# Supplementary material for: Reanalysis of the 2000 Rift Valley fever outbreak in Southwestern Arabia
Source: PLoS One. 2020 Dec 14;15(12):e0233279. doi: 10.1371/journal.pone.0233279 (PMC7735616; doi:10.1371/journal.pone.0233279)

PLOS ONE

Reanalysis of the 2000 Rift Valley fever outbreak in Southwestern Arabia

--Manuscript Draft--

**Manuscript Number:**	PONE-D-19-21159
**Article Type:**	Research Article
**Full Title:**	Reanalysis of the 2000 Rift Valley fever outbreak in Southwestern Arabia
**Short Title:**	Rift Valley fever Arabia
**Corresponding Author:**	Compton J. Tucker<br>NASA<br>Greenbelt, Maryland UNITED STATES
**Keywords:**	First Arabian Rift Valley hemorrhagic fever outbreak; satellite data; medical and veterinary surveillance
**Abstract:**	The first documented Rift Valley hemorrhagic fever outbreak in the Arabian Peninsula occurred in northwestern Yemen and southwestern Saudi Arabia from August 2000 to September 2001. This Rift Valley fever outbreak is unique because the virus was introduced into Arabia during or after the 1997-1998 East African outbreak and before August 2000, either by wind-blown infected mosquitos or by infected animals, both from East Africa. A wet period from August 2000 into 2001 resulted in a large number of amplification vector mosquitoes, these mosquitos fed on infected animals, and the outbreak occurred. More than 1,500 people were diagnosed with the disease, at least 215 died, and widespread losses of domestic animals were reported. Using a combination of satellite data products, including 2 x 2 m digital elevation images derived from commercial satellite data, we show rainfall and potential areas of inundation or water impoundment were favorable for the 2000 outbreak. However, favorable conditions for subsequent outbreaks were present in 2007 and 2013, and very favorable conditions were also present in 2016-2018. The lack of subsequent Rift Valley fever outbreaks in this area suggests that Rift Valley fever has not been established in mosquito species in Southwest Arabia, or that strict animal import inspection and quarantine procedures, medical and veterinary surveillance, and mosquito control efforts put in place in Saudi Arabia following the 2000 outbreak have been successful. Any area with Rift Valley fever amplification vector mosquitos present is a potential outbreak area unless strict animal import inspection and quarantine proceedures are in place.
**Order of Authors:**	Compton J. Tucker
	Katherine A. Melocik
	Assaf Anyamba
	Kenneth J. Linthicum
	Shamsudeen F. Fagbo
	Jennifer L. Small
**Additional Information:**	
**Question**	**Response**
**Financial Disclosure**<br><br>Enter a financial disclosure statement that describes the sources of funding for the work included in this submission. Review the submission guidelines for detailed requirements. View published research articles from *PLOS ONE* for specific examples.	AA: U.S. Army Medical Command contract DHA-2018-770 for Global Emerging Infectious Disease Surveillance

Powered by Editorial Manager® and ProduXion Manager® from Aries Systems Corporation

This statement is required for submission and **will appear in the published article** if the submission is accepted. Please make sure it is accurate.<br><br>**Unfunded studies**<br>Enter: *The author(s) received no specific funding for this work.*<br><br>**Funded studies**<br>Enter a statement with the following details:<br>• Initials of the authors who received each award<br>• Grant numbers awarded to each author<br>• The full name of each funder<br>• URL of each funder website<br>• Did the sponsors or funders play any role in the study design, data collection and analysis, decision to publish, or preparation of the manuscript?<br>• **NO** - Include this sentence at the end of your statement: *The funders had no role in study design, data collection and analysis, decision to publish, or preparation of the manuscript.*<br>• **YES** - Specify the role(s) played.<br><br>* typeset	
**Competing Interests**<br><br>Use the instructions below to enter a competing interest statement for this submission. On behalf of all authors, disclose any competing interests that could be perceived to bias this work—acknowledging all financial support and any other relevant financial or non-financial competing interests.<br><br>This statement **will appear in the published article** if the submission is accepted. Please make sure it is accurate. View published research articles from *PLOS ONE* for specific examples.	The authors have declared that no competing interests exist.

**NO authors have competing interests**<br><br>Enter: *The authors have declared that no competing interests exist.*<br><br>**Authors with competing interests**<br><br>Enter competing interest details beginning with this statement:<br><br>*I have read the journal's policy and the authors of this manuscript have the following competing interests: [insert competing interests here]*<br><br>* typeset	
**Ethics Statement**<br><br>Enter an ethics statement for this submission. This statement is required if the study involved:<br><br>• Human participants<br>• Human specimens or tissue<br>• Vertebrate animals or cephalopods<br>• Vertebrate embryos or tissues<br>• Field research<br><br>Write "N/A" if the submission does not require an ethics statement.<br><br>General guidance is provided below. Consult the submission guidelines for detailed instructions. **Make sure that all information entered here is included in the Methods section of the manuscript.**	N/A

**Format for specific study types**<br><br>**Human Subject Research (involving human participants and/or tissue)**<br>• Give the name of the institutional review board or ethics committee that approved the study<br>• Include the approval number and/or a statement indicating approval of this research<br>• Indicate the form of consent obtained (written/oral) or the reason that consent was not obtained (e.g. the data were analyzed anonymously)<br><br>**Animal Research (involving vertebrate animals, embryos or tissues)**<br>• Provide the name of the Institutional Animal Care and Use Committee (IACUC) or other relevant ethics board that reviewed the study protocol, and indicate whether they approved this research or granted a formal waiver of ethical approval<br>• Include an approval number if one was obtained<br>• If the study involved *non-human primates*, add *additional details* about animal welfare and steps taken to ameliorate suffering<br>• If anesthesia, euthanasia, or any kind of animal sacrifice is part of the study, include briefly which substances and/or methods were applied<br><br>**Field Research**<br><br>Include the following details if this study involves the collection of plant, animal, or other materials from a natural setting:<br>• Field permit number<br>• Name of the institution or relevant body that granted permission	
**Data Availability**<br><br>Authors are required to make all data underlying the findings described fully available, without restriction, and from the time of publication. PLOS allows rare exceptions to address legal and ethical concerns. See the PLOS Data Policy and FAQ for detailed information.	Yes - all data are fully available without restriction

Powered by Editorial Manager® and ProduXion Manager® from Aries Systems Corporation

A Data Availability Statement describing where the data can be found is required at submission. Your answers to this question constitute the Data Availability Statement and **will be published in the article**, if accepted.<br><br>**Important:** Stating 'data available on request from the author' is not sufficient. If your data are only available upon request, select 'No' for the first question and explain your exceptional situation in the text box.<br><br>Do the authors confirm that all data underlying the findings described in their manuscript are fully available without restriction?	
**Describe where the data may be found in full sentences. If you are copying our sample text, replace any instances of XXX with the appropriate details.**<br><br>• If the data are **held or will be held in a public repository**, include URLs, accession numbers or DOIs. If this information will only be available after acceptance, indicate this by ticking the box below. For example: *All XXX files are available from the XXX database (accession number(s) XXX, XXX.).*<br>• If the data are all contained **within the manuscript and/or Supporting Information files**, enter the following: *All relevant data are within the manuscript and its Supporting Information files.*<br>• If neither of these applies but you are able to provide **details of access elsewhere**, with or without limitations, please do so. For example:<br><br>*Data cannot be shared publicly because of [XXX]. Data are available from the XXX Institutional Data Access / Ethics Committee (contact via XXX) for researchers who meet the criteria for access to confidential data.*<br><br>*The data underlying the results presented in the study are available from (include the name of the third party*	All satellite data in our manuscript are available at the Oak Ridge National Laboratory Distributed Active Archive Center (ORNL DAAC).

and contact information or URL).<br>• This text is appropriate if the data are owned by a third party and authors do not have permission to share the data.<br><br>* typeset	
Additional data availability information:	

Powered by Editorial Manager® and ProduXion Manager® from Aries Systems Corporation

Manuscript Click here to download Manuscript Arabian-RVF-2000.docx

PLOS One: 07/10/19 17:33

Reanalysis of the 2000 Rift Valley fever outbreak in Southwestern Arabia

Compton J. Tucker[1*], Katherine A. Melocik[1], Assaf Anyamba[1], Kenneth J. Linthicum[2], Shamsudeen F. Fagbo[3], and Jennifer L. Small[1]

NASA/Goddard Space Flight Center

Earth Sciences Division

Greenbelt, Maryland 20771 USA

[2]U.S. Department of Agriculture

Center for Medical, Agricultural, and Veterinary Entomology

Gainesville, Florida 32608

and

[3]One Health Unit, Executive Directorate for Response and Surveillance,

National Centre for Disease Prevention and Control,

Riyadh, Saudi Arabia

* Corresponding author

E-mail: compton.j.tucker@nasa.gov

Abstract

The first documented Rift Valley hemorrhagic fever outbreak in the Arabian Peninsula occurred in northwestern Yemen and southwestern Saudi Arabia from August 2000 to September 2001. This Rift Valley fever outbreak is unique because the virus was introduced into Arabia during or after the 1997-1998 East African outbreak and before August 2000, either by wind-blown infected mosquitos or by infected animals, both from East Africa. A wet period from August 2000 into 2001 resulted in a large number of amplification vector mosquitoes, these mosquitos fed on infected animals, and the outbreak occurred. More than 1,500 people were diagnosed with the disease, at least 215 died, and widespread losses of domestic animals were reported. Using a combination of satellite data products, including 2 x 2 m digital elevation images derived from commercial satellite data, we show rainfall and potential areas of inundation or water impoundment were favorable for the 2000 outbreak. However, favorable conditions for subsequent outbreaks were present in 2007 and 2013, and very favorable conditions were also present in 2016-2018. The lack of subsequent Rift Valley fever outbreaks in this area suggests that Rift Valley fever has not been established in mosquito species in Southwest Arabia, or that strict animal import inspection and quarantine procedures, medical and veterinary surveillance, and mosquito control efforts put in place in Saudi Arabia following the 2000 outbreak have been successful. Any area with Rift Valley fever amplification vector mosquitos present is a potential

outbreak area unless strict animal import inspection and quarantine proceedures are in place.

Introduction

Rift Valley fever is a mosquito-borne viral hemorrhagic fever that affects domestic animals and humans. First described in 1931, the virus responsible for Rift Valley fever is in the genus *Phlebovirus* and the family *Bunyaviridae*. The first documented occurrence or epizootic of Rift Valley fever occurred in 1930-1931 near Lake Naivasha, Kenya, in the Rift Valley of East Africa, with high mortality among domestic sheep [1]. Although Rift Valley fever primarily affects domestic animals, mainly sheep, goats and cattle, it can also infect humans, causing illness and death in some cases [2]. Most human infections have resulted from bites from infected mosquitoes, through contact with infected animals, and to a lesser extent, through contact with infected animal tissue [3]. Rift Valley fever has been exclusively a disease of Africa and Madagascar, with the exception of the Southwest Arabian 2000-2001 outbreak [4] (Fig 1).

Fig 1. Potential epizootic areas of Rift Valley fever in Africa, Arabia, and Madagascar are shown in green. An insert map of the areas in Saudi Arabia and Yemen where significant numbers of people were infected in the 2000-2001 outbreak. There were no documented Rift Valley fever outbreaks in Arabia prior to 2000-2001.

Rift Valley fever infections in humans are usually mild, with an incubation period of 2 to 5 days following exposure. Those infected may experience no detectable symptoms or may develop a sudden-onset influenza-like fever, headache, gastrointestinal discomfort,

and general body malaise. However, these symptoms are transitory and do not persist for more than 7 days. Other patients develop symptoms that may be mistaken for meningitis: loss of appetite; vomiting; sensitivity to light; and neck stiffness. While most human Rift Valley fever infections are mild, ~4% of human infections are more severe. These more severe infections are manifested as one or more of three syndromes: ocular disease; meningoencephalitis; and/or hemorrhagic fever [2].

While death is uncommon for patients with the ocular form of Rift Valley fever, some infected patients experience a permanent loss of vision. The meningoencephalitis form of Rift Valley fever is usually manifested a few weeks after initial infection, with symptoms including convulsions, lethargy, coma, hallucinations, memory loss, and vertigo. The death rate of this form of the disease is low, although neurological complications can appear several weeks later. The hemorrhagic fever form of Rift Valley fever has a case fatality rate of ~50%. Symptoms of this form of the disease include bleeding in the gastrointestinal tract, bleeding in the skin, bleeding from the nose or gums, other hemorrhage, and severe liver impairment. Death is reported to occur 3 to 6 days after onset of symptoms of this severe form of Rift Valley fever. Fortunately, this Rift Valley fever syndrome is infrequent in infected humans [2].

There is no licensed Rift Valley fever vaccine for humans. Several effective vaccines for animals are available; however, animal immunization must occur before an outbreak starts. Thus, early warning of possible pending outbreaks several weeks in advance is extremely important, as this enables vaccination programs for animals, enables personal protection methods for humans, and enables mosquito vector control to be undertaken.

Mosquito Vector Ecology and Population Dynamics

Rift Valley fever virus transovarially infects the eggs of at least one species of the mosquito genus *Aedes* [5]. That is, the mosquito eggs are laid already infected with the virus. These infected eggs lie dormant in the soil of unflooded mosquito habitats in the endemic area. Following periods of prolonged rainfall, local flooding occurs and the already-infected eggs hatch and immature mosquito stages develop into infected adults. These infected mosquitoes transmit the Rift Valley fever virus to animals upon whom they feed, although usually at a very low level, because they are inefficient transmitters of the disease [6]. Among domestic animals, sheep and cattle are reported to be more susceptible to Rift Valley fever than goats; horses, pigs, and camels ~~are least susceptible~~. Although larger wild animals become infected and develop viremia, most wild animals in endemic regions exhibit minimal disease [7, 8].

Without prolonged rainfall for several weeks, any initial Rift Valley fever outbreak would be local in nature and would not ~~be a reported~~ as outbreak. However, if excessive rainfall continues for 5 to 7 weeks duration or longer, mosquitoes of the genus *Culex* develop in these flooded areas, hatch, become numerous, and are very efficient transmitters of the Rift Valley fever virus (Fig 2). The *Culex* and *Mansonia* species are the amplification or expansion vectors for Rift Valley fever outbreaks. They acquire the virus from infected livestock and then efficiently infect large numbers of other animals quickly [4,9,10].

Fig 2. Mosquito numbers after mosquito habitats were flooded in East Africa as determined from collecting and identifying mosquito species [5].

Rift Valley fever has occurred intermittently over much of sub-Saharan Africa, with outbreaks reported in many countries (Fig 1). In East and Southern Africa, Rift Valley fever activity has been reported in the proximity of grassland depressions [11-14]. These depressions can flood seasonally and, when flooded, are good habitats for the development of immature *Aedes* and *Culex* mosquito species that serve as Rift Valley fever vectors [9,10]. In dry areas of Senegal and Mauritania and in irrigated areas of Egypt, Rift Valley fever outbreaks have occured in riverine areas [15-17].

Area of Study

Our area of study was the southwestern part of the Arabian Peninsula, where Saudi Arabia borders Yemen along the Red Sea (Fig 3). There are three climatic zones present in this region: the Tihama, the dry coastal plain with elevations below 400 meters above sea level and minimal precipitation, usually <150 mm/yr; a region composed of hills with elevations between 400 and 600 meters with <300 mm annual precipitation; and a mountainous region with elevations 600 meters and above with annual precipitation of 300 to 800 mm annual precipitation [18]. The Tihama is a large alluvial plain that is intensely cultivated where irrigation is possible from many dams. Periods of higher precipitation in the Sarawat Mountains result in higher runoff into ephemeral streams that flow to the Tihama. These agricultural areas and reservoirs create pools of standing

water ideal for mosquito production, particularly during periods of excessive and persistent rainfall upstream.

Fig 3. The Landsat-7 NDVI difference between May/June 2000 and September 2000 shows the increase in green vegetation density from increased rainfall by September 2000 in the Sarawat Mountains for southwest Arabia. This in turn, resulted in more runoff to the coastal plain or Tihama that provided ideal conditions for the emergence of Rift Valley fever mosquito vectors. Specific outbreak locations occurred from Al Qunfuda, Saudi Arabia in the north to Wadi Mawr, Yemen in the south and are noted by red triangles.

Remote sensing surveillance of Rift Valley fever is based upon: (1) Monitoring through time rainfall or a surrogate for rainfall in the Rift Valley fever endemic area (Fig 1); and (2) Identifying periods of anomalous rainfall that persist for more than 5 to 7 weeks in the Rift Valley fever endemic area. Previous work using satellite and ground data has documented and confirmed this approach for several areas [10,11,13,19].

We use the satellite normalized difference vegetation index (NDVI) [20] as a surrogate for rainfall. We also introduce a new capability for mapping mosquito breeding habitats, through the use of digital elevation data derived from commercial satellite data stereo image pairs [21]. This new mapping of possible mosquito breeding areas at the 2 x 2 m scale identifies potential inundation areas.

There are two methods commonly used to monitor or infer rainfall from Rift Valley fever endemic areas: (1) Measure rainfall directly using the rain gauge network and/or use rainfall estimates from satellite data such as the Tropical Rainfall Monitoring Mission

[22], geostationary MeteoSat satellites [23], or the Global Precipitation Mission [24] or (2) Use a NDVI remote sensing approach from which rainfall can be inferred [25-27]. While it would be desirable to monitor rainfall directly, the rain gauge network in much of sub-Saharan Africa is sparse and discontinuous [27] and estimates from Meteosat, the Tropical Rainfall Monitoring Mission, and the Global Precipitation Mission have only been available since the early 1990s and have a coarse spatial resolution. For these reasons, and especially for spatial specificity at the scale of tens to hundreds of meters, we use the time series NDVI-rainfall monitoring approach.

NDVI time series exist from the Advanced Very High Resolution Radiometer (AVHRR) instruments since July 1981 globally at an 8-km resolution [28]; from the Satellite Pour l'Observation de la Terre (SPOT) vegetation-PROBA V-Sentinel 3 satellite continuum and have been available globally since May 1998. SPOT vegetation data have a spatial resolution of 1 km from 1998-2013 [29] while PROBA V and Sentinel-3 data are available at 330 m from 2013 [30]; from NASA's moderate resolution imaging spectrometer (MODIS) instruments availably globally since January 2000 at a 250 m resolution [31]; and from Landsat at 30 m since Landsat-5 was launched in 1984 [32].

The NDVI approach we employ is based upon spectral characteristics of vegetation directly related to photosynthesis in terrestrial vegetation. Green leaves in plant canopies have low red reflectance due to strong chlorophyll absorption in the red portion of the spectrum and a very high reflectance in the near infrared portion, where minimal absorption occurs. This unique spectral response of green leaves makes it

possible to differentiate green vegetation from other surface constituents by remote sensing. The normalized difference vegetation index is calculated as:

$$NDVI = \frac{r_{nir} - r_{red}}{r_{nir} + r_{red}} \qquad (1)$$

Where ρ_{nir} and ρ_{red} are the surface reflectances in the near infrared and red portions of the electromagnetic spectrum, respectively [20]. Derived NDVI values range between -1 to +1, with values below zero indicating absence of green vegetation and positive values progressively showing more green leaf density. The variability of rainfall and the dynamics of vegetation in semi-arid lands are a major determinant of life cycles of animals and insects. There is a close relationship between seasonal green vegetation density and persistence, the NDVI, and breeding and upsurge patterns of particular insects, including locusts and mosquitoes [11,12,14,15,33,34].

In addition to using NDVI data from a variety of different instruments to infer rainfall from subsequent green vegetation development, we employ digital elevation images derived from commercial satellite data along-track stereo image pairs to map the potential for inundation at the 2 x 2 m scale in Rift Valley fever outbreak areas [21]. The area of possible inundation is directly related to potential mosquito breeding density.

The Southwestern Arabian 2000 Rift Valley Fever Outbreak

Reports of an outbreak of an unknown disease first appeared in Saudi Arabian newspapers in early September 2000. Initial reports in mid-September 2000 were from ~~Jizan~~ state, in Southwestern Saudi Arabia adjacent to Yemen, and described an unknown

disease infecting ~ 60 people with ~30 fatalities [35]. Disease symptoms in the affected patients were described as high fever, diarrhea, burst arteries, and renal failure. Bleeding from the mouth, nose, and ears and eye lesions were common in affected patients. ~~Jizan~~ state is known for its heat and humidity during the rainy season at this time of year, that makes it an excellent breeding area for mosquitoes. A spokesman for the Saudi Arabian health authority ~~reported~~ the disease was transmitted by sheep and a large number of domestic animals had died in ~~Jizan~~ state, Saudi Arabia [35]. ~~Jizan~~ state lies 300 km across the Red Sea from Africa and is adjacent to Yemen.

Saudi Arabian authorities in ~~Jizan~~ state imposed emergency measures after cases of the Rift Valley fever were confirmed in late 2000. Measures included closing schools for several weeks, intensive mosquito control spraying, and a ban on the importation of domestic animals from the Horn of Africa.

On September 20, 2000, another Rift Valley fever outbreak was reported in the Wadi Mawr area of the Huseidah region of Yemen that resulted in 50 deaths [36]. Laboratory confirmation of the disease in both Saudi Arabia and Yemen was made by the World Health Organization’s Collaborating Centre at the Centers for Disease Control and Prevention in Atlanta, Georgia [37].

By early November 2000, more than 1,500 people were diagnosed as having contracted Rift Valley fever in both countries and at least 215 died: 106 in Saudi Arabia and 109 in Yemen. The death rate is perhaps the highest case fatality rate (~13%) for a Rift Valley fever outbreak to date. However, the number of deaths could be correct while the number of infected people was under reported, thus lowering the case fatality rate.

Several thousand domestic animals also perished but exact numbers were not reported [38].

Materials and Methods

We used three sources of normalized difference vegetation index satellite for our rainfall analysis in southwestern Arabia: 8-km data from 1981 to 2016 derived from the AVHRR instruments carried on a series of polar-orbiting meteorological satellites operated by the National Oceanic and Atmospheric Administration and the European Organization for the Explotation of Meteorological Satellites; NASA 250 m MODIS imagery from 2000 to the present; and NASA and US Geological Service 30 m data derived from Landsat-7's Enhanced Thematic Mapper instrument for 2000 and 2001. We now are able to complement Landsat 30 m data with "harmonized" Landsat-8 and Sentinel-2a and Sentinel-2b 30 m data if greater spatial detail is needed [39]. These harmonized data provide time series NDVI data at a 30 m spatial resolution with a revisit frequency of 3.7 days at the equator. Fig 4 shows the NDVI correspondence between AVHRR 8 km and MODIS 250 m data for the Arabian outbreak area ~~(Fig 4)~~.

Fig 4. (A). The AVHRR NDVI 8 km anomalies from July 1981 to December 2016 from the area in (4c). (B). The MODIS 250 m NDVI anomalies for the same area in (4c) from Al Qunfunda, Saudi Arabia to Wadi Mawr, Yemen, from the coast to the foothills of the Sarawat mountains, an area of 100,000 km^2 in Southwestern Arabia bordering the Red Sea in the image of (C). Positive NDVI values are a consequence of rainfall and are color-coded in blue in both 4a and 4b [26]. Wetter periods always mean more mosquitos in

these areas. Dryer periods are color-coded in red. Note the wetter period from 1994 to 1998 that overlapped with the 1997-1998 Rift Valley fever outbreak in East Africa. It is possible the introduction of Rift Valley fever virus into Southwest Arabia occurred in 1997-1998, while the East Africa 1997-1998 Rift Valley fever outbreak was underway. Note the very similar NDVI trends from 2000 to 2016 derived from AVHRR NDVI and from MODIS NDVI data. The MODIS NDVI data in Fig 4b show favorable conditions for Rift Valley fever outbreaks in 2016-2018 but none have occurred in Arabia during this time.

We also used two DigitalGlobe WorldView-1 along-track stereo pair images acquired on November 7, 2017 only seconds apart from which elevation data were determined. These data were processed using the SETSEM code of [21] to produce a 2 x 2 m digital elevation image with a vertical uncertainty of ±70 cm (Fig 5). An analysis of the digital elevation image for Fig 5 found 2.1 km^2 of potentially inundated area that was comprised of 4,188 individual areas. This capability is a new tool to address mosquito-linked infectious diseases and provides direct identification and innumeration of small mosquito breeding areas.

Fig 5. (A) WorldView-1 panchromatic 2 m resolution image from November 7, 2017 from the Wadi Mawr village area of Northwest Yemen; and (B) a stereo digital elevation image and elevation profile produced from a stereo pair of WorldView-1 panchromatic images acquired on November 7, 2017 from the same area. The total potential water impoundment areas in Fig 5b are represented by red colors, totaled 2.1 km^2, and were comprised of 4,188 different 100 km^2 or greater non-connected areas. Mapping

impoundment areas to identify potential mosquito-breeding habitats is a new tool for infectious disease study.

Results and Discussion

In tandem with the Landsat-7 NDVI analysis in Fig 3, we examined NDVI3g AVHRR data from 1981 to 2016 and MODIS 8-day NDVI from April 2000 through April 2019 (Fig 4). Because the outbreak occurred in September 2000, it must have rained in the region during the preceding weeks to allow for the flooding of mosquito breeding habitats and subsequent emergence of the Rift Valley fever vectors to initiate a large-scale outbreak [4]. The Landsat, NDVI3g, and MODIS NDVI anomalies from May to September 2000 showed a strip of persistent positive anomalies along the Sarawat mountain range. This mountain range, ~2000 meters above the sea and parallel to the Red Sea, is the most prominent elevated topographic feature in this region. Rainfall falling on the mountain range drains into ephemeral streams that in turn flow to the coastal plain. The reported focal areas of the outbreak in Saudi Arabia, Al Qunfuda and ~~Jazan~~ are cities, while Asir is a state. The reported focal area of the outbreak in Yemen was Wadi Mawr, also a district. These four locations are located on the coastal plain (Fig 3).

It has been ~~suggested~~ the 2000 Arabian Rift Valley fever outbreak resulted from the importation of infected livestock animals from East Africa before the 2000 outbreak. The period of rainfall from late August 2000 into 2001 would have provided the conditions for the presence of mosquito amplification vectors. (Fig 4) [40-43]. However, it is not known if the virus was introduced to Arabia in 1997-1998 or 1999-2000.

However, the genetic sequence of the Rift Valley fever viruses isolated in Saudi Arabia during this period were closely related to virus samples isolated during the 1997–1998 epizootic in East Africa [44-46]. While no Rift Valley fever outbreaks were reported in Africa in the June to September 2000 time period, the disease can be present and be undetected in a residual manner within herbivore populations.

Most agriculture in this region is practiced in the coastal plain, and during the heavy rains in the Sarawat Mountains, agricultural fields become inundated, creating ideal habitats for mosquito breeding as we show with our 2 m scale digital elevation surface mapping. Malaria has been known to be endemic in the lowlands of this region and various species of *Anopheles* mosquitoes, including *Anopheles* ~~*arabiens*~~ *and Anopheles gambiae,* have been identified there [47].

Other mosquito species possibly implicated in Rift Valley fever Southwest Arabian outbreak include *Culex tritaneniorhynchus* and *Aedes vexans arabiensis* mosquitoes in the Jizan area of Saudi Arabia, where more than 75,000 mosquitoes were collected between September 25, 2000 and October 10, 2000 and 23,699 were tested for Rift Valley fever virus [48].

Since early 2001, the ~~Ministry of Agriculture~~ of Saudi Arabia has taken precautionary measures to prevent renewed outbreaks of Rift Valley fever. These measures include: providing Rift Valley fever animal vaccines and veterinarians to carry out the vaccination program; undertaking mosquito control efforts during periods of high rainfall; and routine mosquito testing for the Rift Valley fever virus [49]. Medical surveillance of humans has also been actively undertaken, with possible Rift Valley fever cases reported

in September 2004 in Al Karbous village [50], in December 2008 in Asir state [51,52], in May 2009 where 33 cases were reported in Jeddah and 103 in Mecca [53], and in May 2010 in Najran City [54]. Routine veterinary serosurveillance is also undertaken, with five seropositive Rift Valley fever cases detected in four flocks of sheep reported in April 2004 in Jizan, although without clinical confirmation [55].

Al-Afaleq et al. 2012 [56] report a summary of veterinary surveillance, where a total of 3,480 sheep, goats, cattle and camels with no history of vaccination against Rift Valley fever were tested at random in Saudi Arabia. All animals tested were negative for IgG class antibodies against the virus, except three out of 913 goats and four out of 1,508 sheep, which tested positive. No virus activity evidence was found in the areas studied where the three goats and four sheep tested positive. All seven animals were clinically normal. These rare positive cases were attributed to false positives or vaccinated animals transferred from other areas [56].

While Saudi Arabia has instituted a thorough, continuing, and apparently successful program of medical and veterinary Rift Valley fever surveillance since the 2000 outbreak, the same cannot be said for Yemen. The on-going conflict in Yemen has made animal importation inspections, quarantine, and medical and veterinary surveillance activities there impossible since 2015.

Conclusions

The Arabian 2000 Rift Valley fever outbreak was unique in that infected animals were imported into areas where large numbers of mosquito amplification vectors were

present, although it is not known when the infected animals were introduced into Arabia. Amplification vector mosquitos fed upon infected animals and the outbreak started. Using satellite data, we document the 2000 to 2018 precipitation regime for the coastal areas to the Sarawat Mountains of southwest Arabia with time series MODIS NDVI data at the 250 m scale in Fig 4. These data, coupled with the extensive number of potential-inundated mosquito breeding areas determined from digital elevation imagery at the 2 x 2 m scale in Fig 5, document excellent mosquito breeding potential in southwest Arabia in wetter periods. The fact no major Rift Valley fever outbreaks have occurred since 2000, and especially that none have occurred from 2015 to 2018 when the rainfall regime was very favorable to outbreaks, leads to two conclusions: (1) the Rift Valley fever reservoir was not established in *Aedes* mosquitos in Southwest Arabia after the 2000 outbreak; and/or (2) effective Rift Valley fever domestic animal quarantine and import restrictions, medical and veterinary surveillance, and mosquito control institutued by the Saudi Arabian Ministry of ~~Agriculture~~ have prevented outbreaks in that country. The conflict in Yemen since 2015 prevents any actions like those undertaken in Saudi Arabia and is cause for concern.

Acknowledgments

This work was supported by a U.S. Army Medical Command contract DHA-2018-770 for Global Emerging Infectious Disease Surveillance. We wish to acknowledge the assistance of Mohammed Al-Hazmi from the Ministry of Health, Jizan, Saudi Arabia and Sami Mukhdari Mushta from the Saudi Center for Disease Prevention and Control,

Riyadh, Saudi Arabia for assistance in both understanding the sequence of events in the 2000 Rift Valley fever outbreak and their description of the control and survellience methods within Saudi Arabia that have prevented subsequent outbreaks.

References

1. Daubney R, Hudson JR, Garnham PC. Enzootic hepatitis or Rift Valley fever: An undescribed virus disease of sheep, cattle and man from East Africa. Journal of Pathology and Bacteriology. 1931;34: 545-579.

2. WHO. Rift Valley fever Fact Sheet. Revised February 19, 2018; Https://www.who.int/en/news-room/fact-sheets/detail/rift-valley-fever

3. Peters CJ, Linthicum KL. Rift Valley fever, in Handbook of Zoonoses, G.B. Beran, editor, CRC Press, Inc; 1994; Boca Raton, Florida, pp. 125-138.

4. Linthicum KJ, Britch SC, Anyamba A. Rift Valley Fever: Am emerging mosquito-borne disease. Annual Review of Entomology 2016;61: 395-415.

5. Linthicum KJ, Davies FG, Kairo A, Bailey CL. Rift Valley fever virus (family *Bunyaviridae*, genus *Phlebovirus*). Isolations from *Diptera* collected during an interepizootic period in Kenya. Journal of Hygiene, Cambridge. 1985; 95: 197-209.

6. Turell M, Gargan T, Bailey CL. *Culex pipiens* as a vector of Rift-Valley fever. American Journal of Epidemiology. 1982;116(3): 556-556.

7. Britch SC, Binepal YS, Ruder MG, Kariithi HM, Linthicum KJ, Anyamba A, et al. Rift Valley fever risk map model and seroprevalence in selected wild ungulates and camels from Kenya. Plos One. 2013;8(6): e66626.doi:10.1371/journal.pone.0066626. e0162759.doi:10.1371/journal.pone 0162759.

8. Meegan JM, Bailey CL. Rift Valley fever, in The Arboviruses: Epidemiology and Ecology, Monath TP editor, Vol. IV 1989; CRC Press, Inc; Boca Raton, Florida, pp. 51-76.

9. Linthicum KJ, Davies FG, Bailey CL, Kairo A. Mosquito species succession in a dambo in an east-African forest. Mosquito News. 1983;43(4): 464-470.

10. Linthicum, KJ, Davies FG, Bailey CL, Kairo A. Mosquito species encountered in a flooded grassland dambo in Kenya. Mosquito News 1984;44(4): 228-232.

11. Linthicum KJ, Bailey CL, Davies FG, Tucker CJ. Detection of Rift-Valley fever viral activity in Kenya by satellite remote-sensing imagery. Science 1987;235(4796): 1656-1659.

12. Linthicum KJ, Anyamba A, Tucker CJ, Kelley PW, Myers M, Peters CJ. Climate and satellite indicators to forecast Rift Valley fever epidemics in Kenya. Science. 1999;285(5426): 397-400.

13. Woods CW, Karpati AM, Grein T, McCarthy N, Gaturuku P, Muchiri E et al. An outbreak of Rift Valley fever in northeastern Kenya, 1997–1998. Emerging Infectious Disease 2002;8(2): 138-144.

14. Anyamba A, Linthicum KJ, Mahoney R, Tucker CJ, Kelley PW. Mapping potential risk of Rift Valley fever outbreaks in African savannas using vegetation index time series data. Photogrammetric Engineering & Remote Sensing. 2002; 68(2): 137-145.

15. Linthicum KJ, Bailey CL, Tucker CJ, Gordon SW, Logan TM, Peters CJ, et al. Observations with NOAA and SPOT satellites on the effect of man-made alterations in the ecology of the Senegal River basin in Mauritania on Rift Valley fever virus transmission. Sistema Terra. 1994;3: 44-47.

16. Wilson ML, Chapman LE, Hall DB, Dykstra EA, Ba K, Zeller HG, Traore-Lamizana M, et al. Rift-Valley fever in rural northern Senegal - human risk-factors and potential vectors. American Journal of Tropical Medicine and Hygiene 1994;50(8): 663-675.

17. Meegan JM, Hoogstraal H, Moussa MI. Epizootic of Rift Valley fever in Egypt in 1977. Veterinary Record 1979;105(6): 124-125.

18. Ageel ARM, Amin MA. Integration of schistosomiasis-control activities into the primary health care system in the Gizan regions, Saudi Arabia. Annals of Tropical Medicine and Parasitology. 1997;91(8): 907-915.

19. Anyamba A, Linthicum KJ, Tucker CJ, Small JL, Britch SC, Gaydos J. Remote Sensing Contributions to Prediction and Risk Assessment of Natural Disasters caused by Large Scale Rift Valley Fever Outbreaks. Proc. of the IEEE. 2012;99:http://dx.doi.org/10.1109/JPROC.2012.2194469

20. Tucker CJ. Red and photographic infrared linear combinations for monitoring vegetation. Remote Sensing of Environment. 1979;8(2): 127-150.

21. Noh MJ, Howat IM. The Surface Extraction from TIN based Search-space Minimization (SETSM) algorithm. ISPRS Journal of Photogrammetry and Remote Sensing. 2017;129: 55-76.

22. Simpson J., Kummerow C, Tao WK, Adler RF. On the tropical rainfall measuring mission (TRMM). Meteorology and Atmospheric Physics. 1996;60(1-3): 19-36.

23. Tucker MR, Sear CB. A comparison of Meteosat rainfall estimation techniques in Kenya. Meteorological Applications 2001;8(1): 107-117.

24. Hou AY, Kakar RK, Neeck S, Ardeshir A, Kummerow CD, Kojima M., et al. The Global Precipitation Measurement Mission. Bulletin of the American Meteorological Society. 2013;95(5): 701-722, DOI: 10.1175/BAMS-D-13-00164.

25. Herrmann SM, Anyamba A, Tucker CJ. Recent trends in vegetation dynamics in the African Sahel and their relationship to climate. Global Environmental Change-Human and Policy Dimensions 2006;15(4): 394-404.

26. Tucker CJ, Nicholson SE. Variation in the size of the Sahara Desert from 1980 to 1997. Ambio 1999;28(7): 587-591.

27. Nicholson SE, Davenport ML, Malo AR. A comparison of the vegetation response to rainfall in the Sahel and East Africa, using normalized difference vegetation index from NOAA AVHRR. Climate Change. 1990;17(2-3): 209-241.

28. Pinzon JE, Tucker CJ. A non-stationary 1981-2012 AVHRR NDVI3g time series. Remote Sensing. 2014;doi:10.3390/rs50x000x, July 2014.

29. Saint G. Spot-4 Vegetation system - association with high-resolution data for multi-scale studies. Calibration and Applications of Satellite Sensors for Environmental Monitoring. 1995;17(1): 107-110.

30. Donlon C, Berruti B, Buongiorno A, Ferreira M, Femenias P, Frerick J et al. The Global Monitoring for Environment and Security (GMES) Sentinel-3 Mission. Remote Sensing of Environment 2012;120: 37-57. doi10.1016/j.rse.2011.07.024.

31. Justice CO, Vermote EF, Townshend JRG, Defries R, Roy DP, Hall DK, et al. The Moderate Resolution Imaging Spectroradiometer (MODIS): Land remote sensing for global change research. IEEE Transactions on Geoscience and Remote Sensing. 1998;36(4): 1228-1249.

32. Goward SN, Masek JG, Williams DL, J. Irons JR, and Thompson RJ. The Landsat-7 mission: terrestrial research and applications for the 21st century. Remote Sensing of Environment. 2001;78(1-2): 2-12.

33. Tucker CJ, Hielkema JU, Roffey J. The potential of satellite remote-sensing of ecological conditions for survey and forecasting desert-locust activity. International Journal of Remote Sensing. 1985;6(1): 127-138.

34. Hielkema JU, Roffey J, Tucker CJ. Assessment of ecological conditions associated with the 1980/1981 desert locust plague upsurge in West-Africa using environmental satellite data. International Journal of Remote Sensing 1986;7(11): 1609-1622.

35. Reuters. Rift Valley Fever - Saudi Arabia. ProMED-mail 2000; 17 Sept. 20000917.1592. <http://www.promedmail.org>. Accessed 18 September 2000.

36. Europa Press. Rift Valley Fever - Yemen. ProMED-mail 2000; 20 Sept. 20000920.1625. <http://www.promedmail.org>. Accessed 22 September 2000.

37. Arishi H, Ageel A, Rahman MA, Hazmi AA, et al. Outbreak of Rift Valley fever—Saudi Arabia, August-October, 2000, Morbidity and Mortality Weekly Report. 2000;49(40): 905-908.

38. Fagbo S. Arab News, November 28, 2000. More Rift Valley fever deaths in Asir. ProMED-mail 2000; 30 Nov. 20001130.2088. <http://www.promedmail.org>. Accessed 28 November 2000.

39. Li J, Roy DP. A global analysis of Sentinel-2A, Sentinel-2B and Landsat-8 data revisit intervals and implications for terrestrial monitoring. Remote Sensing. 2017;9: 902; doi:10.3390/rs9090902.

40. Little PD, Teka T, Azeze A. Cross-Border Livestock Trade and Food Security in the Horn of Africa: An Overview. 2001; (Washington DC, US: USAID/REDSO), 27 p.

41. Salem SA, Tran A, Grosbois V, Gerbier G, Al-Qadasi M, Saeed K, et al. Can environmental and socioeconomic factors explain the recent emergence of Rift Valley fever in Yemen, 2000-2001? Vector-Borne and Zoonotic Diseases. 2011a;11(6): 773-780.

42. Salem SA, Waret-Szkuta A, Roger F, Olive M-M, Saeed K, Chevalier, V. Risk assessment of the introduction of Rift Valley fever from the Horn of African to Yemen via legal trade of small ruminants. Tropical Animal Health Production. 2011b;42: 471-480.

43. Balkhy HH, Memish ZA. Rift Valley fever: an uninvited zoonosis in the Arabian Peninsula. International Journal of Antimicrobial Agents. 2003;21: 153-157.

44. Shoemaker T, Boulianne C, Vincent MJ, Pezzanite L, Al-Qahtani MM, Al-Mazrou Y, et al. Genetic analysis of viruses associated with emergence of Rift Valley fever in Saudi Arabia and Yemen, 2000-2001. Emerg Infect Dis. 2002;8(12): 1415–1420.

45. Miller BR, Godsey MS, Crabtree MB, Savage HM, Al-Mazrao Y, Al-Jeffri MH

et al. Isolation and genetic characterization of Rift Valley fever virus from *Aedes vexans arabiensis*, Kingdom of Saudi Arabia. *Emerg Infect Dis*. 2002;8(12): 1492-1494.

46. Samy AM, Peterson AT, Hall M. Phylogeorgraphy of Rift Valley fever virus in Africa and the Arabian Peninsula. PLOS One Negl Trop Dis 2017;11(1): e0005226. doi:10.1371/journal.pntd.0005226

47. Jupp PG, Kemp A, Grobbelaar A, Leman P, Burt FJ, Alahmed AM, et al. The 2000 epidemic of Rift Valley fever in Saudi Arabia: mosquito vector studies. Medical and Veterinary Entomology. 2002;16(3) 245-252.

48. Haddad O. Malaria: clinical aspects and treatment. Annals of Saudi Medicine. 1990;15(4): 348-348.

49. Arab News, August 21, 2003. Ministry takes steps to prevent RVF outbreak. ProMED-mail 2003;20031063.2489.

50. Saudi Gazette, September 27, 2004. Rift Valley fever – Saudi Arabia (Jizan). ProMED-mail 2004; 20040928.2685.

51. Saudi Gazette, December 3, 2008. Rift Valley fever – Saudi Arabia (Asir), Suspected, Request for information. ProMED-mail 2008;20081203.3798.

52. Saudi Gazette, December 16, 2008. RVF detected in Asir region. ProMED-mail 2008;20081203.3894.

53. Middle East online, May 31, 2009. Rift Valley Fever –Saudi Arabia alert. ProMED-mail 2009;20090602.2053.

54. Fagbo S. May 6, 2010. Rift Valley fever virus infection of a resident of Najran. ProMed-mail 2010;20100507.1485.

55. OIE Disease Information October 1, 2004 vol. 17(40); ProMed-mail 2004.20041003.2723.

56. Al-Afaleq AI, Hussein MF, Al-Naeem AA, Housawi F, Kabati AG. Seroepidemiological study of Rift Valley fever in animals in Saudi Arabia. Tropical Animal Health Production. 2012;44: 1535-1539.

Author Contributions

Conceptulization: CJT AA KJL

Data Curation: KAM JLS

Formal Analysis: CJT KAM JLS AA KJL

Funding acquisition: AA

Investigation: CJT KAM AA KJL JLS

Resources: CJT AA KAM KJL JLS

Software: KAM JLS

Supervision: CJT

Validation: KAM JLS SFF

Visualization: CJT KAM JLS SFF

Writing original draft: CJT

Writing – review & editing: CJT AA KJL KAM JLS SFF

Data Avaliability Statement: All satellite data are available at the Oak ridge National Laboratory Distributed Active Archive Center (ORNL DAAC).

Funding: The work was supported by the Department of Defense Health Agency-Armed Forces Health Surveillance Branch (AFHSB) Global Emerging Infections Surveillance and Response System (GEIS) under Project # P0019_18_NS (AA). The funders had no role in the study design, data collection and analysis, decision to publish, or preparation of the manuscript.

Competing Interests: The authors declare that we have no competing interests.

 Click here to download Figure Fig5a-b.jpg

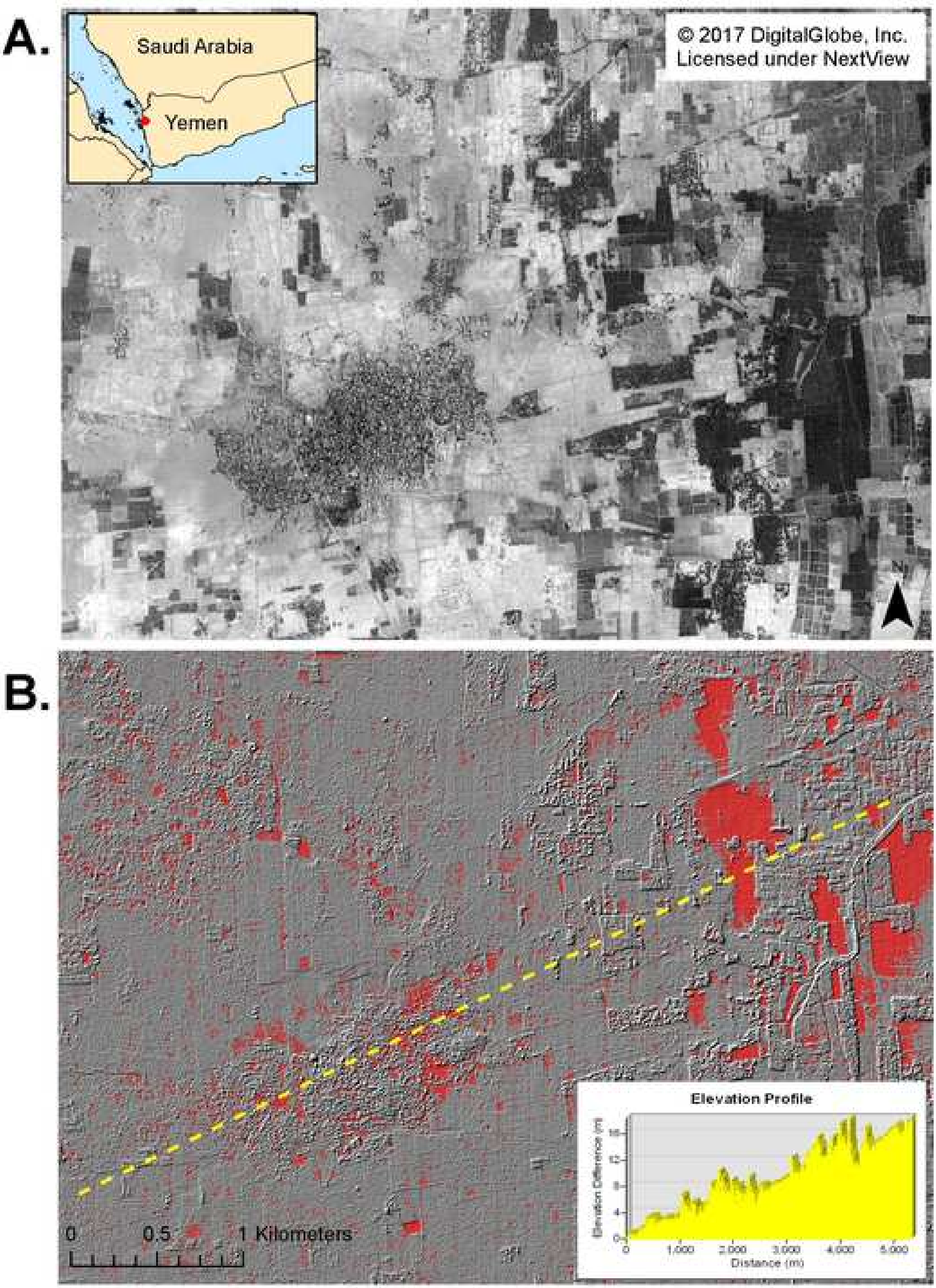

Figure

Click here to download Figure Fig4.psd

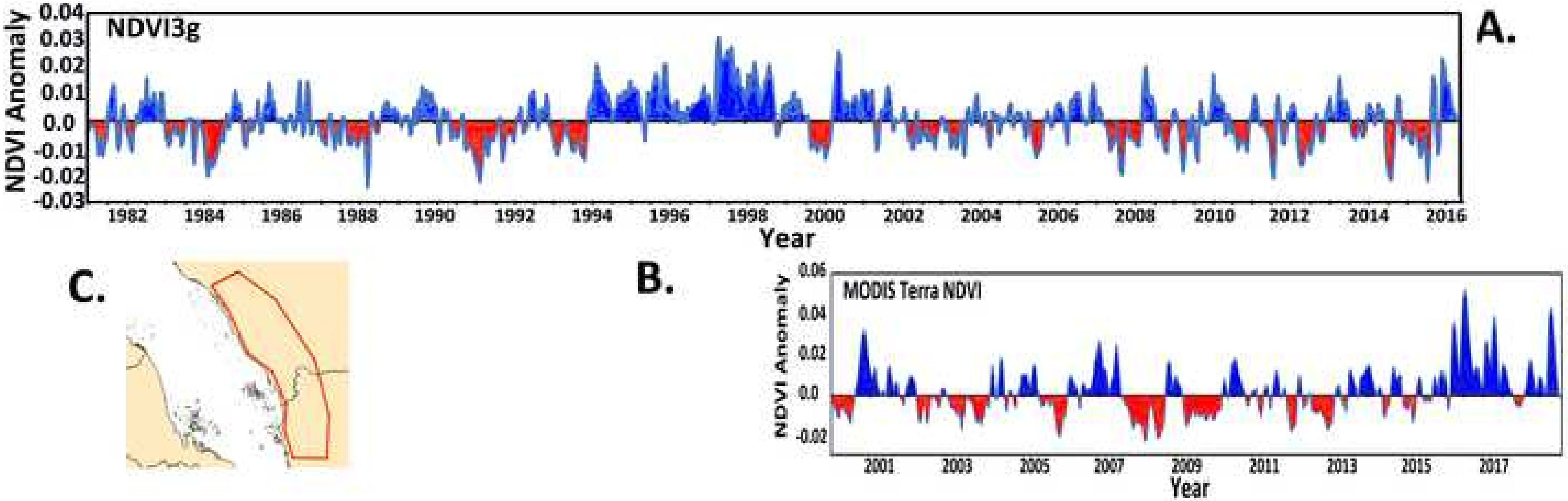

Figure

Click here to download Figure Fig3.psd

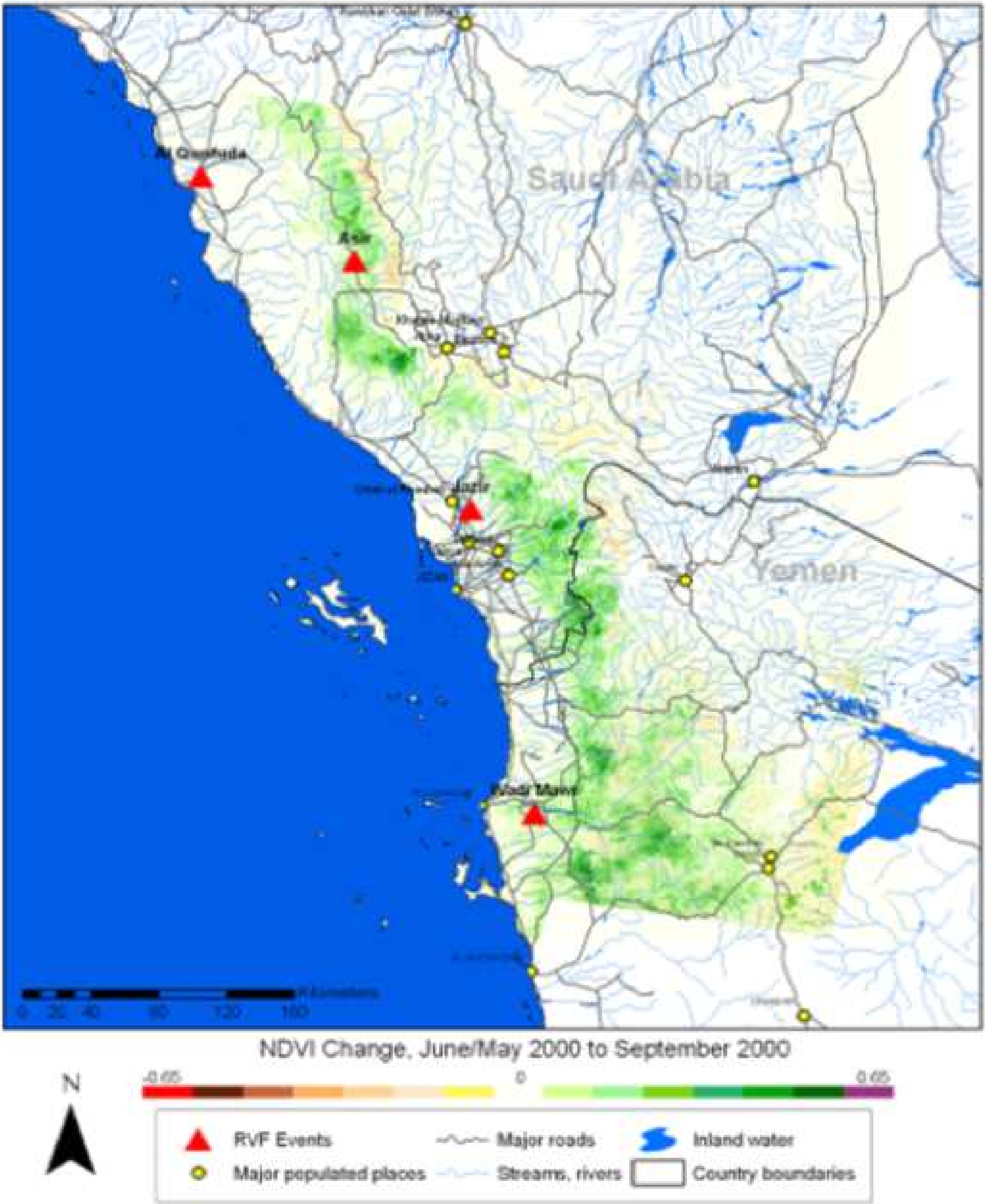

Figure

Click here to download Figure Fig2.psd

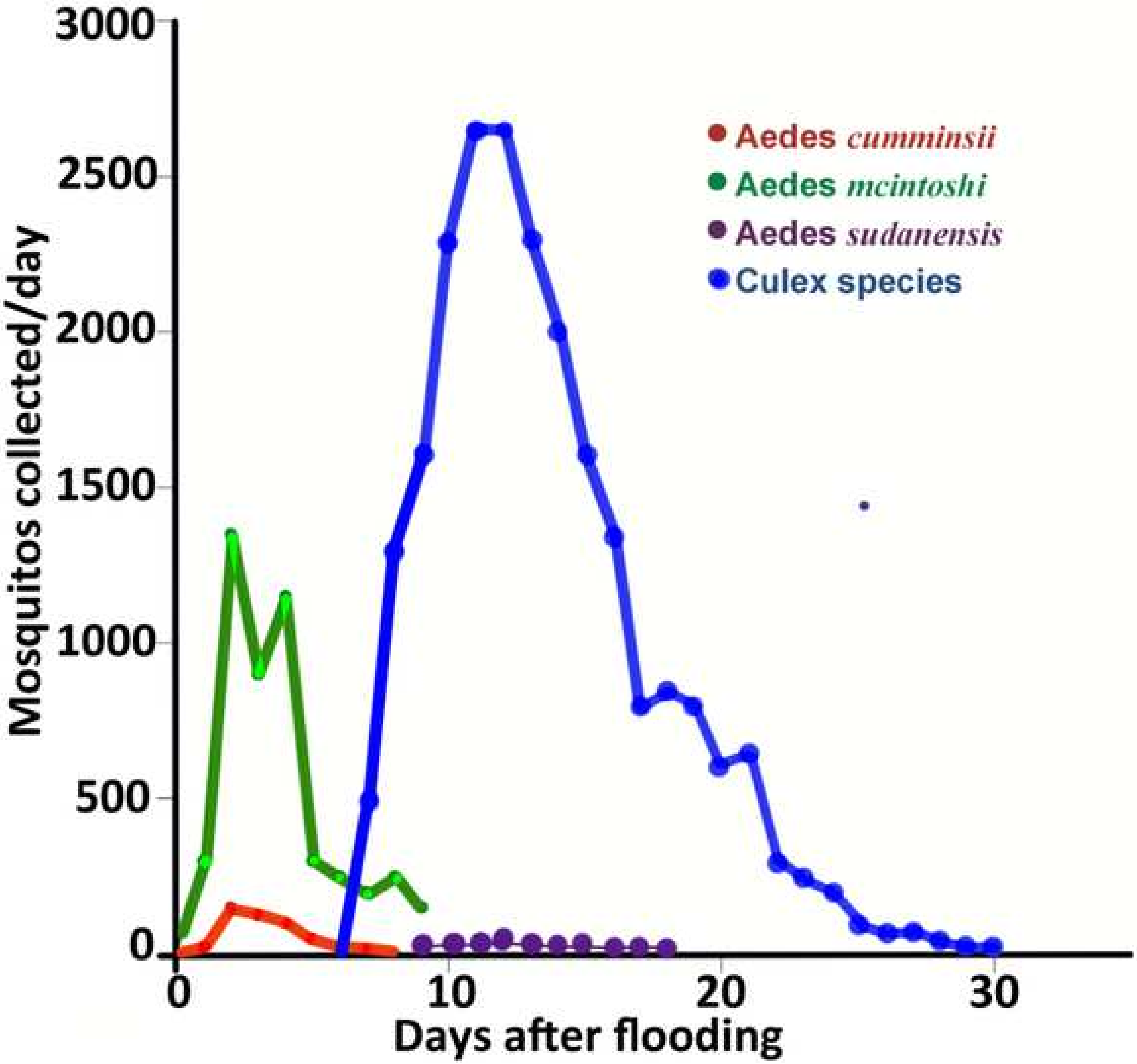

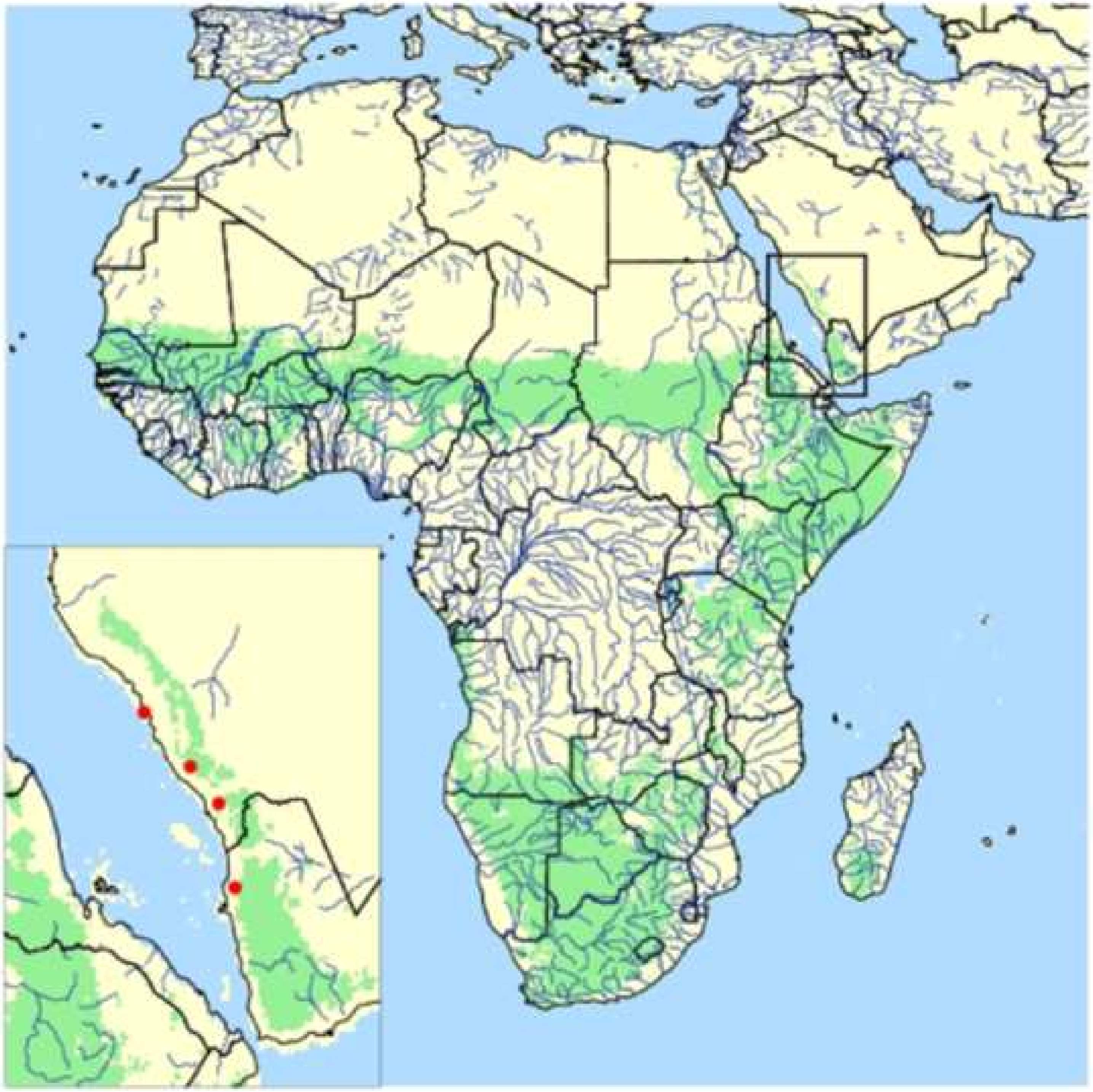

Supplement: Attachment — Submitted filename: REV-PONE-D-19-21159_reviewer.pdf [file pone.0233279.s001.pdf]
