# Supplementary material for: Reanalysis of the 2000 Rift Valley fever outbreak in Southwestern Arabia
Source: PLoS One. 2020 Dec 14;15(12):e0233279. doi: 10.1371/journal.pone.0233279 (PMC7735616; doi:10.1371/journal.pone.0233279)
Supplement: Attachment — Submitted filename: Response to Reviewers PONE-D-19-21159.docx [file pone.0233279.s002.docx]

We agree with all the points and suggestions of the reviewers and have addressed every one of their concerns or questions:

1. We have studied PLOS ONE's style requirements and have modified our manuscript accordingly;

2. Figures 1 and 3 are figures which Jennifer Small, one of our coauthors, produced. Because she is employed by NASA, these images are in the public domain and are not copyrighted. Katherine Melocik, another coauthor produced figure 5. Figure 5a is copyrighted and we have obtained permission from the National Geospatial Intelligence Agency for public release of figure 5a--it is labeled accordingly. Because figure 5b is a derived product from NASA-licensed commercial satellite data, this figure is not copyrighted.

3. All data used in our manuscript are publicly available. This includes the Landsat-7 data in figure 3, the AVHRR data in figure 4a, the MODIS data in figure 4b, figure 5a, and the digital DEM in figure 5b.

4. We have amended our list of authors ensuring that each author is linked to an affiliation.

Reviewers' comments:

Reviewer #1: We have heeded this reviewers comments and suggestions for every one of his nine numbered points. We thank this reviewer for his thoroughness.

Reviewer #2: We replaced line 94 with exactly the wording suggested by this reviewer. We also have made excellent progress using satellite data to predict Rift Valley fever virus outbreaks in areas such as Kenya and South Africa to allow vaccination of animals prior to outbreaks.

Reviewer #3: We use 2 m WorldView-1 satellite data in figure 5 because these data are a stereo pair. These data enable the 2 m scale 3-D mapping of inundation potential for mosquito breeding habitats. We do not have Rift Valley fever data at the same spatial scale. However, our point here is to show a new potential for mapping fine-scale mosquito breeding habitats.

We thank reviewer #3 for bringing to our attention the work of Samy, Kenawy, and Townsend (2014). Our NASA librarian was able to find a description of this poster from the 2014 ASTMH meeting. We concur with reviewer #3 that this reference is very similar to our work and cite this work accordingly as reference 39 in our revised manuscript. We give this reference credit for their earlier description of the methodology for what we have also done. Furthermore, we acknowledge that our work strengthens their earlier work by using additional satellite data available after 2014.

We were able to scale different spatial resolutions of diverse climate data by using 30 m Landsat data as in figure 3, compared to the 250 m MODIS data and 8 km AVHRR data we used in figure 4.

We acknowledge Samy, Kenawy, and Townsend (2014) are the masters of ecological niche modeling which we did not use. Instead, we focused on uses of satellite data that support their expertise in ecological niche modeling. We showed very similar NDVI results using Landsat, MODIS, and AVHRR data sets over a 38-year period at three different spatial scales. Lastly, we show the use of stereo satellite data to map inundation potential at the 2 m scale. This technique compliments ecological niche modeling where inundation is an important consideration for ecological niches.

We attempted to use robust validation tools to test our Arabian predictions. See lines 292 to 315 in our revised manuscript. However, the results of these validations were mixed and inconclusive. This led us to conclude that Saudia Arabia had instituted a thorough and continuing program of medical and veterinary Rift Valley fever surveillance since the 2000 outbreak.
